# Retinal Inhibition of CCR3 Induces Retinal Cell Death in a Murine Model of Choroidal Neovascularization

**DOI:** 10.1371/journal.pone.0157748

**Published:** 2016-06-16

**Authors:** Haibo Wang, Xiaokun Han, Deeksha Gambhir, Silke Becker, Eric Kunz, Angelina Jingtong Liu, M. Elizabeth Hartnett

**Affiliations:** 1 The John A. Moran Eye Center, University of Utah, Salt Lake City, Utah, United States of America; 2 Department of Ophthalmology, The Fourth Affiliated Hospital of China Medical University, Shenyang, P.R. China; Indiana University College of Medicine, UNITED STATES

## Abstract

Inhibition of chemokine C-C motif receptor 3 (CCR3) signaling has been considered as treatment for neovascular age-related macular degeneration (AMD). However, CCR3 is expressed in neural retina from aged human donor eyes. Therefore, broad CCR3 inhibition may be harmful to the retina. We assessed the effects of CCR3 inhibition on retina and choroidal endothelial cells (CECs) that develop into choroidal neovascularization (CNV). In adult murine eyes, CCR3 colocalized with glutamine-synthetase labeled Műller cells. In a murine laser-induced CNV model, CCR3 immunolocalized not only to lectin-stained cells in CNV lesions but also to the retina. Compared to non-lasered controls, CCR3 mRNA was significantly increased in laser-treated retina. An intravitreal injection of a CCR3 inhibitor (CCR3i) significantly reduced CNV compared to DMSO or PBS controls. Both CCR3i and a neutralizing antibody to CCR3 increased TUNEL+ retinal cells overlying CNV, compared to controls. There was no difference in cleaved caspase-3 in laser-induced CNV lesions or in overlying retina between CCR3i- or control-treated eyes. Following CCR3i, apoptotic inducible factor (AIF) was significantly increased and anti-apoptotic factor BCL2 decreased in the retina; there were no differences in retinal vascular endothelial growth factor (VEGF). In cultured human Műller cells exposed to eotaxin (CCL11) and VEGF, CCR3i significantly increased TUNEL+ cells and AIF but decreased BCL2 and brain derived neurotrophic factor, without affecting caspase-3 activity or VEGF. CCR3i significantly decreased AIF in RPE/choroids and immunostaining of phosphorylated VEGF receptor 2 (p-VEGFR2) in CNV with a trend toward reduced VEGF. In cultured CECs treated with CCL11 and/or VEGF, CCR3i decreased p-VEGFR2 and increased BCL2 without increasing TUNEL+ cells and AIF. These findings suggest that inhibition of retinal CCR3 causes retinal cell death and that targeted inhibition of CCR3 in CECs may be a safer if CCR3 inhibition is considered as a therapy for neovascular AMD.

## Introduction

Neovascular age-related macular degeneration (AMD) is a leading cause of vision loss and legal blindness in individuals over 60 years of age [1]. Current therapies include anti-angiogenic agents and sometimes, anti-inflammatory and photodynamic therapies, administered as monotherapy or in combination [2]. Of these treatments, agents targeting vascular endothelial growth factor (VEGF) are the most widely used. Intravitreal injection of anti-VEGF agents is effective at inhibiting choroidal neovascularization (CNV) in about 40% patients, but long-term risks include potential retinal and ocular harm from loss of VEGF-beneficial effects and rare complications related to intraocular injections [3,4]. Therefore, ancillary treatments are being considered.

The G-protein coupled receptor (GPCR), C-C chemokine receptor 3 (CCR3) has been studied extensively for its role in leukocyte biology and is known to be a target in pulmonary allergic conditions [5,6]. Also, some studies have provided evidence to support CCR3 inhibition as a treatment for neovascular AMD [6]. The mechanisms whereby CCR3 inhibition affects angiogenesis are incompletely understood. We previously found crosstalk between VEGF and CCR3 signaling, suggesting that inhibitors of CCR3 may not only function by affecting CCR3-induced CNV, but also by inhibiting CCR3 interactions with VEGF-induced CNV [7]. We also found that CCR3 expression occurred not only in choroidal endothelial cells (CECs) but also in the neural retina of eyes from aged donors. Therefore, we sought to determine whether broad inhibition of CCR3 signaling to inhibit CNV would be safe for retinal cell survival.

In this study, we addressed the hypothesis that broad retinal inhibition of CCR3 using an intravitreal injection to deliver a CCR3 inhibitor induces apoptosis of cells, thereby permitting CEC death, but also potentially adversely affects retinal health. We used in vitro methods and the laser-induced CNV model in mouse to address the hypothesis. We provide evidence that CCR3 inhibition does not directly lead to caspase-3-induced CEC apoptosis but may work by inhibiting downstream signaling pathways through VEGF receptor 2 (VEGFR2). Furthermore, in the setting of a CCR3 ligand and VEGF, CCR3 inhibition induces Műller cell apoptosis through a caspase 1/3 independent mechanism involving apoptotic inducible factor (AIF). In the laser-induced CNV model, broad CCR3 inhibition, i.e, by intravitreal delivery, reduced survival of retinal cells overlying CNV lesions. These findings support additional study regarding safety of CCR3 inhibitors and the consideration of CEC-targeted CCR3 inhibition.

## Materials and Methods

### Animals

C57/Bl6 wild-type male and female mice (Jackson Laboratory, Bar Harbor, ME) were used in the laser-induced CNV model. Anesthesia was obtained with ketamine (100 mg/kg) and xylazine (10 mg/kg). The care and use of animals in our studies adhered to the University of Utah guidelines (*Guide for the Care and Use of Laboratory Animals*) and Association for Research in Vision and Ophthalmology Statement for the Use of Animals in Ophthalmic and Vision Research.

### Laser-induced CNV Model and Intravitreal Injections

For the laser-induced CNV model, 6–8 week old C57Bl/6 mice were used, as recommended by Lambert et al [8]. Both eyes were dilated with one drop of 1% tropicamide ophthalmic solution (Bauch & Lomb, Rochester, NY, USA). Mice were anesthetized, placed onto a heated stage and then treated with 4–5 spots of 532 nm laser photocoagulation, each about 2 disc diameters from the optic nerve, with the Phoenix Image-Guided Laser System 94 (Phoenix Micron IV, Pleasanton, CA) at settings of 400 mW intensity and 100 ms duration that caused cavitation bubbles confirming disruption of Bruch’s membrane. Following laser injury, 1 μL intravitreal injections of 10 μg/μL CCR3 receptor inhibitor, SB328437 [6] (CCR3i, Calbiochem, Billerica, MA) vs. DMSO control or a neutralzing antibody against mouse CCR3 (CCR3Ab, 1 μg/μl; R&D Systems, Minneapolis, MN) vs. isotype IgG (R&D Systems) were performed in both eyes of mice. The same treatment (experimental or control) was given to both eyes of each animal to avoid confounding from possible crossover effects.

### Optical Coherence Tomography

Image-guided spectral domain coherence tomography (sdOCT, Phoenix Labs) was used to visualize and measure the length and the height of CNV lesions. Three to 5 lesions from 6–8 different animals per condition were imaged at the thickest region and captured for analysis, using the software available on the Phoenix OCT (Phoenix).

### Retinal Flat-mount Processing, Imaging and Analysis

After euthanasia, eyes were enucleated, and fixed in 4% paraformaldehyde (PFA) for 2 hours at room temperature (RT). Posterior eyecups consisting of RPE/choroid/sclera were dissected, blocked in 1% Triton-X with 5% normal goat serum (NGS) and then stained overnight at 4°C with AlexaFluor (AF) 568-conjugated isolectin B4 (1:200) (Molecular Probes, Euguene, OR). Eyecups were then flattened by cutting radial incisions and flat-mounted. Z-stack imaging was performed at 20X using the Olympus confocal microscope (FV1000, Olympus, Melville, NY) and a 3 μm step size. Lesions were excluded from imaging if they were not round or merged into one another or if there was blood. Integrated fluorescence from stacked images of each lesion was measured and the outcomes from images summed to calculate lesion volumes (μm^3^) for experimental and control treatments. Lesion volumes were then averaged to obtain a single value for each condition. At least 40 spots from 10 different flat-mounts were analyzed for each treatment.

### Immunostaining in Retinal Cryosections

Three or seven days after laser-induced injury, eyes were harvested for immunostaining. Briefly, eyes were enuclated, fixed in 4% PFA for 1 hour, and then embedded in optimal cutting temperature (OCT) and sectioned (Tissue Tek, Hatfield, PA). For immunofluorescence, cryosections (12 μm) were first blocked in 0.1% Triton-X with 5% normal goat serum (NGS) and then stained overnight at 4°C with the following primary antibodies: rabbit anti-cleaved caspase-3 (1:100; Cell Signaling Technology, Boston, MA); rabbit anti-p-VEGFR2 (Y951) antibody (1:100;Cell Signaling Technology, Beverly, MA); rabbit anti-CCR3 antibody (1:50; Santa Cruz Technology) or rabbit anti-glutamine synthetase (1:100; BD Transduction Laboratories, San Jose, CA). The following day, sections were washed three times and incubated with AlexaFluor (AF) 488-conjugated goat anti-rabbit antibody (1:200; Life technologies, Grand Island, NY) for p-VEGFR2 or cleaved caspase-3 and AF 568-conjugated isolectin B4 (1:500) to identify CNV lesions or with Topro3 to label the nuclei (1:500; Life Technologies, Grand Island, NY) for 1 hour at RT. Sections were then counterstained with DAPI Fluoromount-G mounting media or Fluoromount-G mounting media without DAPI for sections stained with Topro 3 (SouthernBiotech, Birmingham, Alabama). Pancreatic tissue sections from 6-week-old wild-type mice provided positive controls for cleaved caspase-3. Rabbit IgG labeling instead of primary antibody was used as a negative control for all experiments. Densitometry for cleaved caspase-3 and phosphorylated-VEGF receptor 2 (p-VEGFR2) labeling was performed using ImageJ software. At least 3–5 retinal cryosections from 3–5 different eyes were imaged for each treatment.

### Cell Culture and Treatments

Human primary CECs were isolated from male and female human donor eyes obtained from the Utah Lions Eye Bank as described previously [9]. CECs were grown in endothelial growth medium (EGM2; Lonza, Walkersville, MD) and used between passages 3 to 5. For each condition in an experiment, at least 2 samples were analyzed and each experiment was repeated twice. Experimental findings were confirmed using different donor eyes.

MIO-M1cells, a human Müller glia cell line, were purchased from E-Lucid (London, United Kingdom). Cells were cultured in DMEM, high glucose, GlutaMAX™ Supplement with pyruvate (Life Technologies, Grand Island, NY) and 10% fetal calf serum (FCS).

Treatments were performed in confluent cells. Before treatment, CECs were starved in serum and growth factor free endothelial basal medium (EBM2; Lonza, Walkersville, MD) and MIO-M1 cells were fasted in DMEM, high glucose, GlutaMAX™ Supplement with 2% FCS overnight. Each cell type was then pre-treated with a small molecule CCR3 inhibitor (CCR3i) (100 nM; a gift of Axikin Pharmaceuticals, San Diego, CA) that prevents ligand-receptor binding, or a VEGFR2 tyrosine-kinase inhibitor (SU5416, 5 μM; Sigma-Aldrich, St. Louis, MO) for 1 hour followed by incubation with CCL11 (10 ng/ml; Axikin Pharmaceuticals) or recombinant VEGF (20 ng/ml; R&D systems, Minneapolis, MN) overnight. DMSO vehicle was used as a control for the CCR3i and SU5416, and PBS as a control for CCL11 and VEGF.

### TUNEL Assay in Retinal Cryosections and in Cultured Cells

TUNEL assays were performed per the manufacturer’s instructions (In Situ Cell Death Kit, TMR red; Roche Diagnostics, Indianapolis, IN). Sections incubated with DNase I (3000 U/ml in 50 mM Tris-HCl, pH 7.5, 1mg/ml BSA) for 10 minutes at 15–25°C were used as positive controls. To identify TUNEL+ cells, cryosections were first labeled with AlexaFluor (AF) 488-conjugated isolectin B4 (1:200; Molecular Probes, Euguene, OR), Iba-1 (1:100; ABCAM, Cambridge, MA), Thy-1 (1:100; ABCAM), or glutamine synthetase, and then with TUNEL. For TUNEL staining in cultured cells, cells were plated on cell culture coverslips (Thermoscientific, Rochester, NY). After staining, images were taken using a fluorescence microscope with five random images per section or coverslip. TUNEL-positive (+) cells were counted in each retinal section from lasered and non-lasered eyes injected with CCR3i vs. DMSO or PBS control or from laser-eyes treated with CCR3Ab *vs*. IgG control. In cultured cells, TUNEL+ cells determined by colabeling with DAPI stained nuclei were quantified and the mean of TUNEL+ cells in the five images from the same coverslip was used for comparison. There were at least 3–5 retinal cryosections or coverslips per condition.

### Protein Preparation and Western Blot Analysis

RPE-choroid isolates from enucleated eyes 7 days after laser in CCR3i and control groups were separated from neural retinas and homogenized in RIPA buffer. MIO-M1 cells or CECs with different treatments were also homogenized in RIPA buffer. Sample protein concentration was measured using the Bicinchocinic acid (BCA) assay (Pierce Biotechnology, Rockford, Illinois). Twenty μg of protein from each treatment was loaded into NuPAGE 4% to 12% Bis-Tris Gels (Invitrogen, Carlsbad, CA), transferred to a PVDF membrane and PVDF membranes were then blocked in 5% milk at RT and then incubated with rabbit anti-caspase-3 antibody (1:1000; Cell Signaling), rabbit anti-BCL2 (1:1000; Cell Signaling), rabbit anti-apoptotic inducing factor (AIF) (1:1000; Cell Signaling), rabbit anti-brain derived neurotrophic factor (BDNF) (1:500, Santa Cruz Technology), phosphorylated VEGFR2 (Y951, 1:500, Santa Cruz Technology) or rabbit anti-VEGF (1:500, Santa Cruz Technology) overnight at 4°C. On the following day, membranes were washed 3X and incubated with HRP-linked goat anti-rabbit secondary antibody (1:3000, Santa Cruz Technology) for 1 hour at RT. Membranes were washed again, placed into West Pico Chemiluminescent Substrate (Thermo Scientific, Rochford, IL) and developed. Beta-actin (Santa Cruz Technology, 1:3000) was used as a loading control. Samples from 4–6 different animals for each treatment were analyzed.

### RNA Isolation and Real Time Quantitative PCR (Real-time qPCR)

CCR3 mRNA was measured in RPE-choroid isolates of non-lasered C57/Bl6 mice at various ages and in neural retinas from lasered animals using quantitative real-time PCR (n = 3-5/group). Briefly, eyes were enucleated and dissected to procure either RPE-choroids or retina. Tissues were homogenized in Tri-reagent (Sigma) and RNA purified from the tissues was analyzed for RNA concentration using NanoDrop 1000 (Thermoscientific, Wilmington, DE). Two μg of RNA was then reverse-transcribed to cDNA using TaqMan Reverse Transcription kit (Applied Biosystems, Foster City, CA) following the manufacturer’s protocol. Real-time qPCR amplifications were run using detection chemistry (SYBR Master Mix, Roche, Indianapolis, IN). GAPDH was as an internal control. Each PCR was run in triplicate and repeated three times. Sequences for the primers were: mouse CCR3: Forward 5^’^-CCAGCTGTCAGCAGAGTAAA-3^’^, Reverse 5^’^- CTCACCAACAAAGGCGTAGA-3^’^; mouse GAPDH: Forward 5^’^-GGAGAAACCTGCCAAGTATGA -3^’^, Reverse 5^’^-TCCTCAGTGTAGCCCAAGA -3^’^.

### Statistical Analysis

Student’s t-test (for non-skewed data) or Mann Whitney U test (for skewed data) was used when comparing data from two means. One-Way ANOVA was performed for experiments having three or more means with Tukey’s HSD (honest significant difference) post-hoc test. A p-value <0.05 was considered statistically significant. Data are presented as means ± SEM or SD as indicated in the Figure legends. For animal studies, 6–10 individual mice were analyzed for CNV volume (n = 4 spots/eye), Micron IV imaging and optical coherence tomography. For TUNEL and other immunohistochemical labeling, 3–4 retinal sections at 60 μm intervals were taken from one eye of 3–5 aninals in each group. For protein analyes of RPE/choroid or retina, western blots were performed from one eye of 4–6 different mice for each treatment. For *in vitro* studies, each experimental condition included an n = 6 from three independent experiments.

## Results

### CCR3 expressed within CNV lesions and laser-treated retinas

**Fig 1:**
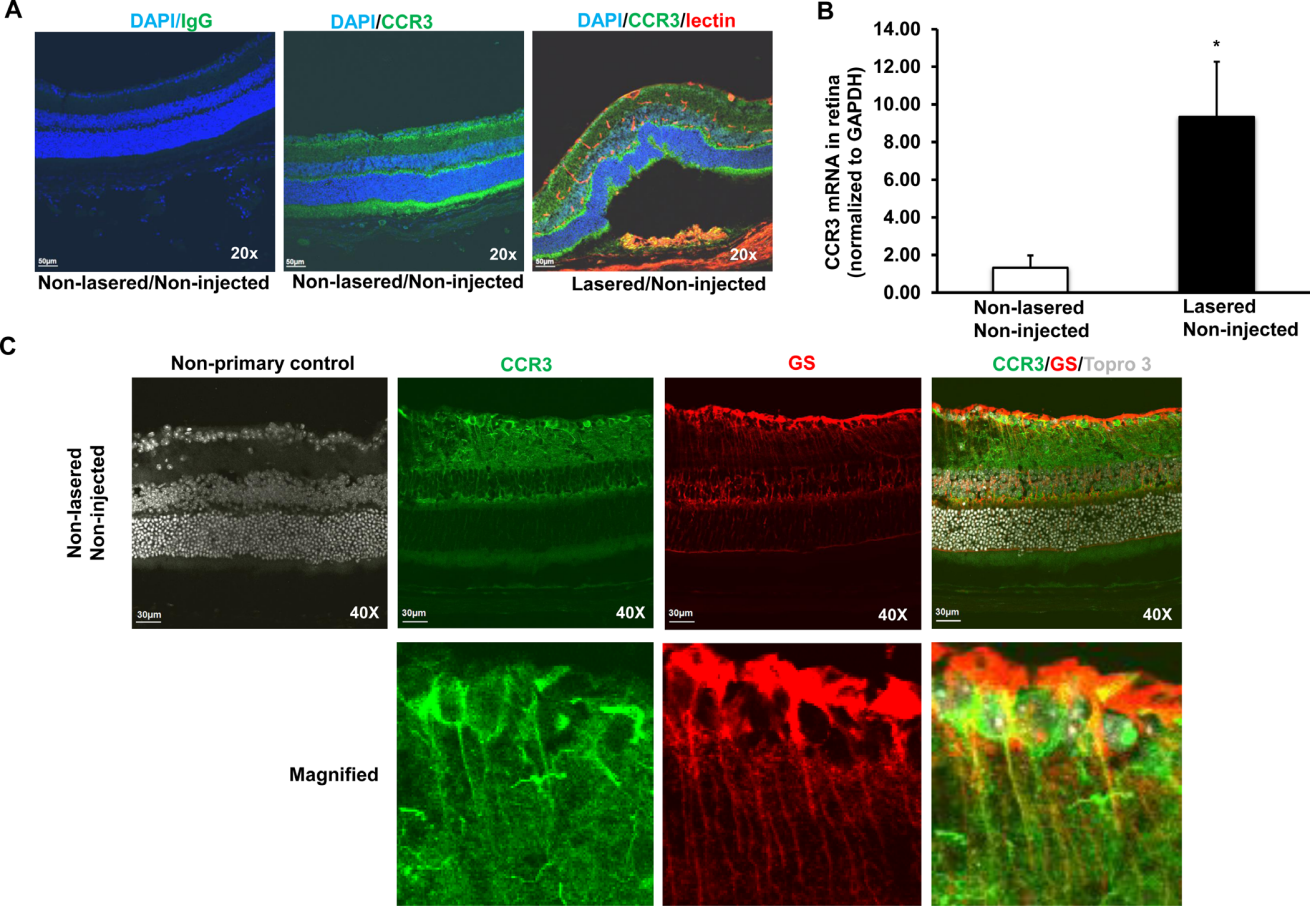
CCR3 is expressed in the retina and at CNV lesions, and upregulated in laser-treated retina. (A) Immunolabeling of CCR3 in retinal cryosections from non-lasered mice without injection (Non-lasered/Non-injected) and from mice 7 days after laser without injection (Lasered/Non-injected) mice (Mag, 20X; Scale bar, 50 μm; Green, CCR3; Blue, DAPI; Red, lectin). (B) Real-time qPCR of CCR3 mRNA in the retina without RPE/choroids from Non-lasered/ Non-injected and Lasered/Non-injected mice (*p<0.05 *vs*. Non-lasered/Non-injected; Results were Mean±SEM; *n =* 4–6 samples per group). (C) Co-immunolabeling of CCR3 and glutamine synthetase in retinal cryosections from Non-lasered/Non-injected adult mice (Upper row-Mag, 40X and lower row-Magnified images; Scale bar, 50 μm; Green, CCR3; Red, glutamine synthetase; Gray, Topro3)

The laser-induced model of CNV shares features to neovascular AMD in that both involve acute wound-healing events [10] and have similar activated signaling mechanisms [11,12]. The model results in increased generation of reactive oxygen species (ROS) [13], angiogenic factors and inflammatory compounds [14], including VEGF [15] and CCL11 [7]. These broad categories of oxidative stress, angiogenesis, and inflammation are implicated in the pathophysiology of neovascular AMD. In retinal cryosections from non-lasered, non-injected 6–8 week old mouse eyes (non-lasered/non-injected), CCR3 immunolabeling was found in retinal ganglion cell and inner and outer nuclear layers and colabeled with glutamine-synthetase (Fig 1A and 1C). In lasered/non-injected eyes, CCR3 colabeled with lectin-stained endothelial cells within CNV lesions 7 days after laser (Fig 1A). Compared to non-lasered/non-injected retinas (without RPE/choroid), CCR3 mRNA was significantly increased in lasered/non-injected eyes (Fig 1B).

### CCR3 inhibition effectively reduces CNV width in the laser-induced model

**Fig 2:**
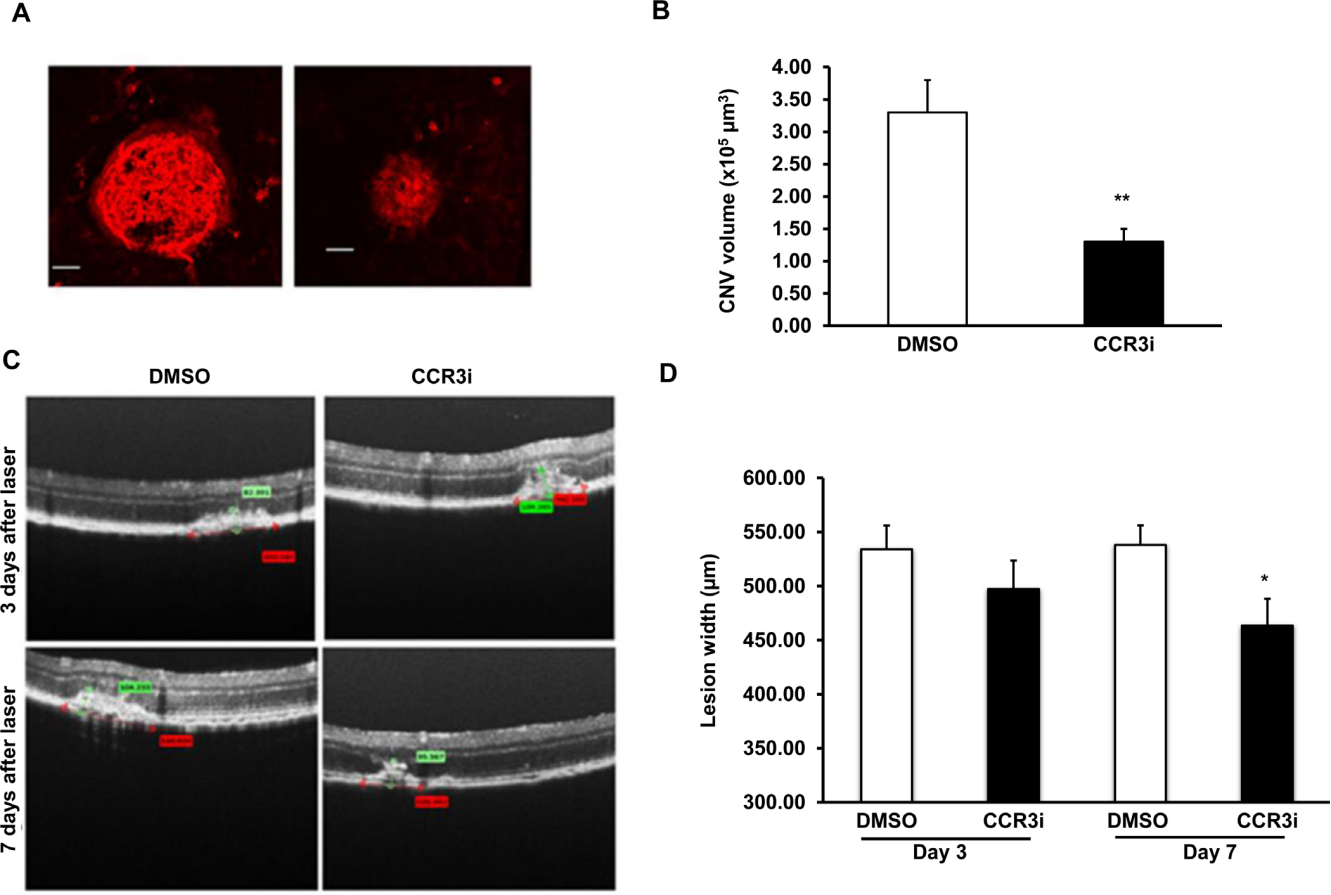
Inhibition of CCR3 effectively reduces CNV in the laser-induced model. Six-eight week old C57Bl/6 mice were lasered and administered 1 μL intravitreal CCR3i (10 μg/μl) or DMSO: (A) and (B) CNV volumes calculated 7 days after laser in isolectin stained RPE/choroidal flatmounts (Mag, 20X; Scale bar, 50 μm) (A, representative flatmount images and B, quantification of CNV volume; n = 40 spots/ group; **p<0.01 *vs*. DMSO treated eyes). (C) and (D) Lesion widths (red caliper) calculated 3 and 7 days after laser injury in DMSO and CCR3i groups (C, representative flat mount images and D, quantification of lesion width; n = 20–24 lesions/data point;**p<0.01 *vs*. day 7 DMSO).

CCR3 inhibition has been reported to decrease laser-induced CNV in a dose-dependent manner [6,16,17]. We confirmed a dose dependent relationship with the CCR3i used (data not shown) and determined that at a dose of 10 μg of CCR3i delivered as a 1 μL intravitreal injection, CNV volume was significantly reduced compared to control 7 days after laser (Fig 2A and 2B). There was also a pattern of decreased CNV width in the CCR3i group at day 3 and a significant decrease in width at day 7 (Fig 2C and 2D). No differences were noted in lesion heights in both groups at either time point (data not shown).

### Increased cell death with CCR3 inhibition in the retina overlying CNV lesions

**Fig 3:**
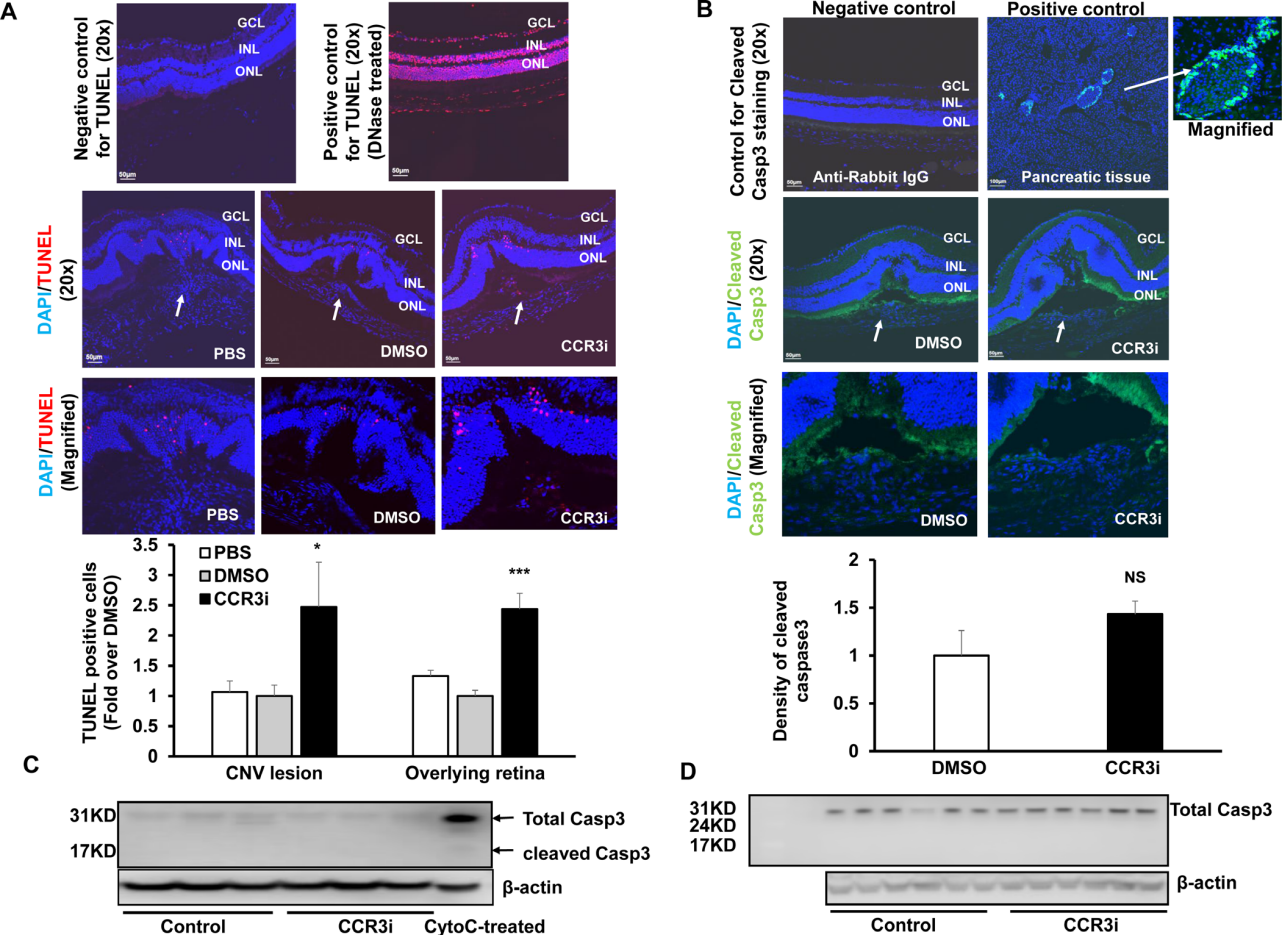
Increased cell death from CCR3 Inhibition in the retina overlying CNV independent of activated caspase-3. (A) TUNEL assay and quantification of TUNEL+ cells on retinal sections from 7 days after laser in PBS, DMSO or CCR3i treated eyes; DNase-I treated sections were positive controls (Red, TUNEL; Blue, DAPI; Upper two rows-Mag, 20X, Scale bar, 50 μm and lower row-Magnified images). Number of TUNEL+ cells (white arrows) in lasered eyes within lesions or adjacent non-lasered areas was counted. Mann-Whitney U test used for statistical analysis: *p<0.05 *vs*. DMSO treated eyes; *n* = 3–4 sections/eye from 3–5 different animals. (B) Immunostaining of cleaved caspase-3 on retinal sections from 7 days after laser in DMSO or CCR3i treated eyes (Upper two rows-Mag-Mag. 20X; Scale bar, 50 μm and lower row-Magnified images) and integrated fluorescence density calculated in CNV lesions or retina overlying lesions. Pancreatic sections from age-matched wild-type mice were positive controls and sections incubated with Rabbit IgG were used as negative controls. Student’s t-test used for statistical analysis: p = NS, vs. DMSO treatment. *n* = 3 sections/eye from 3–5 different animals (Green, Cleaved caspase-3; Blue, DAPI). Western blots of cleaved caspase-3 and total caspase-3 in (C) RPE/choroids or (D) retinas from 7 days after laser in DMSO or CCR3i treated eyes (Cell lysates treated with cytochrome C (CytoC treated) were used as a positive control for cleaved caspoase 3).

We previously reported that CCR3 labels the neural retina of aged human donor eyes and in mouse eyes following laser to induce CNV [6,7]. We, therefore, determined whether CCR3 inhibition at a dose that inhibits laser-induced CNV would adversely affect retinal cell survival. First, we found that there was no difference in the number of TUNEL+ cells in the retina of non-lasered eyes injected with either DMSO control or CCR3i (S1A and S1B Fig). We next calculated TUNEL+ cells in the retina overlying laser-induced CNV lesions in DMSO- and CCR3i- injected eyes. As shown in Fig 3A, 7 days after laser, there was a significant increase in TUNEL+ cells within CNV lesions in CCR3i-treated eyes compared to either DMSO- or PBS- treated. (Some TUNEL+ cells colabeled with lectin (S1C Fig)). There were also significantly increased TUNEL+ cells in the retina overlying CNV in CCR3i-treated eyes compared to control DMSO or PBS. However, there was no difference in TUNEL+ cells in CNV lesions or in the retina overlying CNV between PBS and DMSO (Fig 3A). In an additional experiment, TUNEL staining was performed in retinal cryosections from lasered mice treated with an intravitreal neutralizing antibody to mouse CCR3 (CCR3Ab). Compared to IgG control injection, CCR3Ab significantly increased TUNEL+ cells in the retina overlying CNV lesions (S1D Fig). These data provide evidence that CCR3 inhibition not only led to reduced cell survival within lasered regions, but also to reduced survival of cells in the retina overlying CNV.

### Increased cell death by CCR3 Inhibition independent of activated caspase-3

We then determined whether a caspase 3-dependent pathway was involved in CCR3i–induced cell death within CNV lesions by immunolabeling sections or by performing western blots for cleaved caspase-3 in retinas or RPE/choroids from CCR3i-treated and DMSO-treated eyes at several time points after laser. Pancreatic tissue from a wild-type mouse was used as a positive control. Active caspase-3 mildly labeled the retina overlying the CNV lesion in both DMSO and CCR3i groups (Fig 3B). There was no cleaved caspase-3 detected in retinas at 8 hours (data not shown) and no difference in fluorescent density of cleaved caspase 3 staining in CNV lesions or in the retina overlying CNV lesions 7 days after laser from CCR3i-treated eyes or DMSO control (Fig 3B). To further determine if CCR3 inhibition induced cell death through activation of caspase-3, we measured cleaved caspase-3 in RPE/choroidal (Fig 3C) or retinal (Fig 3D) lysates by western blot analyses and found that there was no cleaved caspase-3 in either RPE/choroids or retinas from CCR3i-treated eyes or DMSO control. Taken together, these data did not support the hypothesis that CCR3 inhibition increased cell death through a caspase-3-dependent pathway.

### CCR3 Inhibition promotes cell death events in retina and in cultured human Müller cells

**Fig 4:**
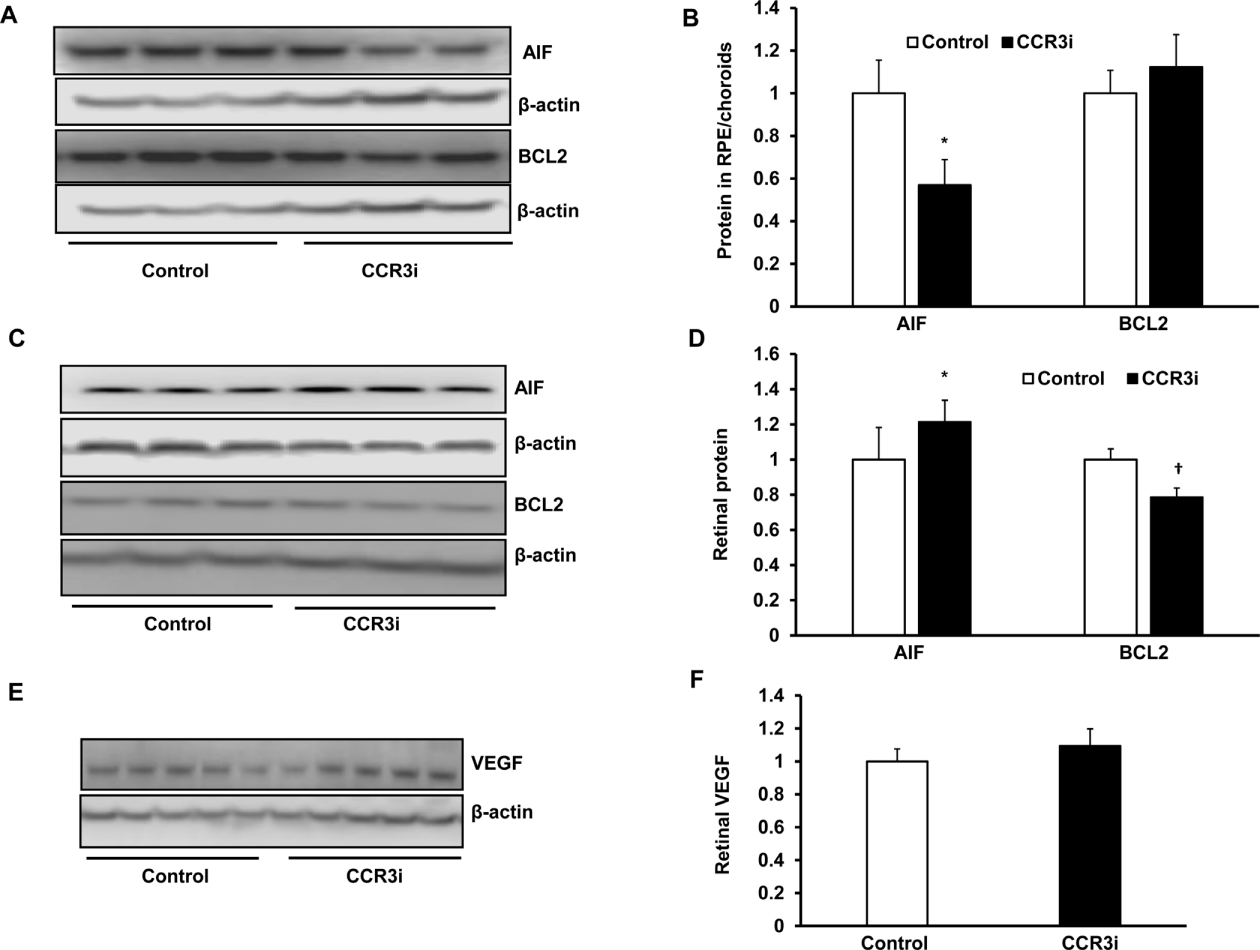
Inhibition of CCR3 promotes cell death events in the retina via a VEGF-independent signaling. Western blots and quantification of densitometry of apoptotic inducing factor (AIF) and anti-apoptotic protein, BCL2, in (A and B) RPE/choroids and (C and D) retinas from DMSO or CCR3i treated eyes 7 days after laser (A and B, reprentative gel images; C and D, quantification of densitometry; *p<0.05 *vs*. Control for AIF protein, ^†^p<0.05 *vs*. Control for BCL2 protein). (E) Western blot and (F) quantification of densitometry of VEGF in retinas from mice 7 days after laser in DMSO or CCR3i treated eyes

We then probed other mechanisms of cell death. Retina and RPE/choroids were harvested separately 7 days after laser from CCR3i- or DMSO-treated mice and processed for apoptotic inducing factor (AIF) and the anti-apoptotic protein, BCL2, by western blot. Compared to DMSO control, CCR3i treatment significantly decreased AIF protein in RPE/choroidal lysates (Fig 4A and 4B), but increased AIF protein and decreased BCL2 protein in retinal lysates (Fig 4C and 4D), suggesting that inhibition of retinal CCR3, but not of RPE/choroidal CCR3, activated apoptotic signaling. Neutralizing or inhibiting retinal VEGF activity can lead to retinal neural and glial degeneration [18,19]. Therefore, we measured VEGF protein in the retina to determine if retinal apoptotic signaling was associated with reduced VEGF. The CCR3i did not affect retinal VEGF protein concentration (Fig 4E and 4F), suggesting that activated apoptotic signaling in CCR3i-treated retina was not related to reduced VEGF expression.

**Fig 5:**
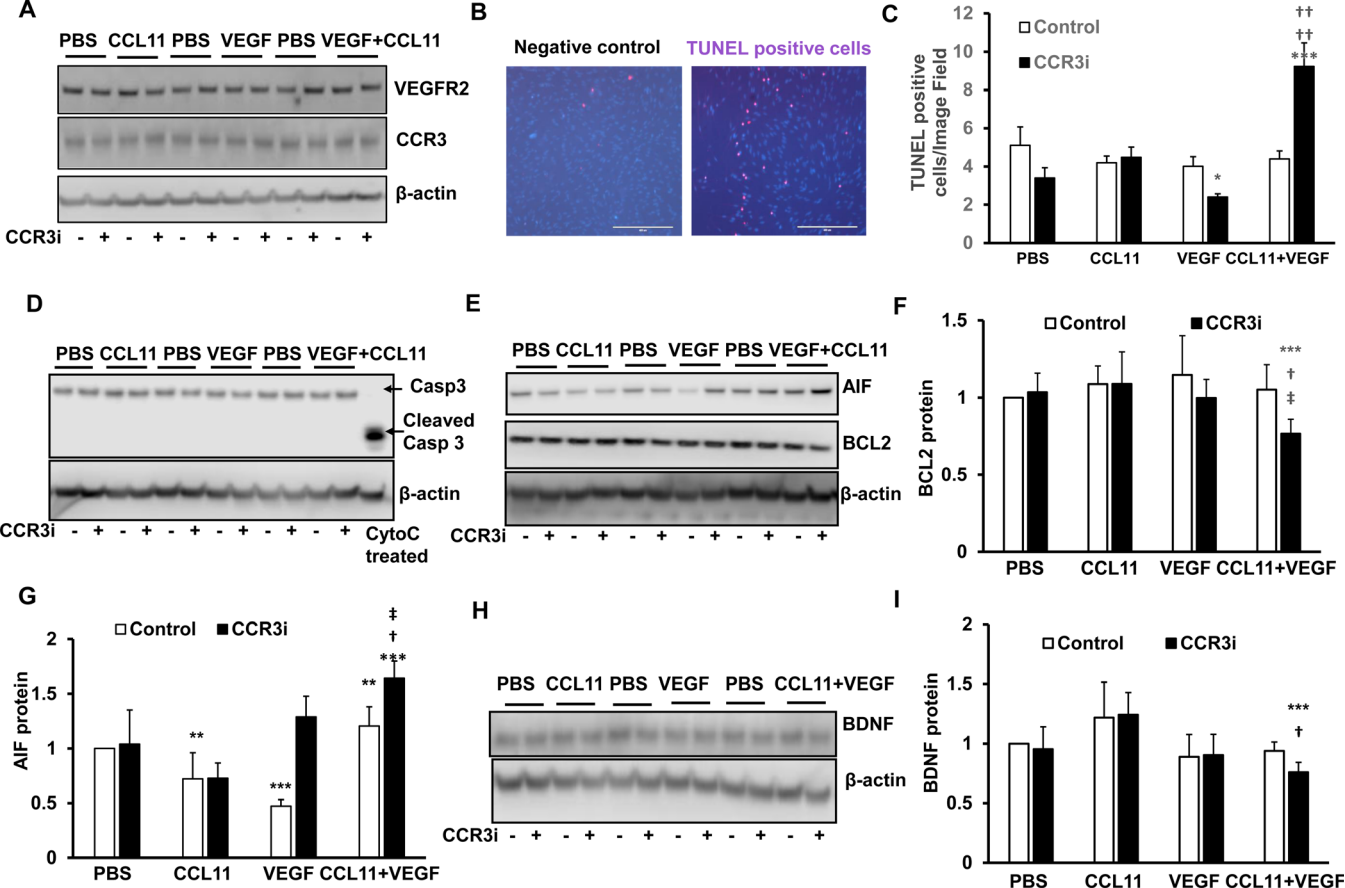
Inhibition of CCR3 promotes cell death events in cultured human Műller cells. In cultured human MIO-M1 cells treated with PBS, CCL11, VEGF, or CCL11 and VEGF with pretreatment with CCR3i or control (DMSO), (A) western blots of CCR3 and VEGFR2. (B) TUNEL staining. (C) Quantification of TUNEL+ cells. (D) Western blots of cleaved caspase-3 and total caspase-3. (E) Western blots and quanfication of (F) BCL2 and (G) of AIF. (H) Western blots and (I) quanfication of BDNF (*p<0.05, **p<0.01, ***p<0.001 *vs*. Control of PBS; ^†^p<0.05, ^††^p<0.01*vs*. CCR3i of PBS; ^‡^p<0.05, ^‡‡^p<0.01 *vs*. control of CCL11+VEGF).

We attempted to identify the TUNEL+ cells in the retina overlying CNV lesions. We colabeled retinal sections with several different cell markers, includling microglia (Iba), Müller cells (glutamine synthetase) ganglion cells (Thy-1) but were unable to identify the TUNEL+ cells overlying and within CNV lesions. Cells undergoing cell death can lose their markers of differentiation. In addition, co-labeling cell compartments using different cell markers can be difficult to discern on immunohistochemical sections. Because co-localization of CCR3 with glutamine synthetase occurred in untreated retinal sections (Fig 1C), we were interested to determine if Müller cells might be one cell type affected by CCR3i. In retinal degenerations including neovascular AMD, proliferation and/or migration of Müller cells, has been reported [20,21]. A recent report demonstrates the presence of GFAP+, vimentin labeling cells on the vitreal surface of human neovascular AMD specimens [21]. In addition to other functions, Müller cells are important in providing trophic support to other retinal neurons [18,19]. We addressed the hypothesis that CCR3i reduced Müller cell survival by culturing the human Müller cell line, MIO-M1 cells, until confluency and exposing them to CCL11, VEGF, CCL11 and VEGF, or control PBS for 24 hours. CCL11 and VEGF are factors previously found to be overexpressed after laser injury to the retina [16,22]. To determine if CCR3 and VEGFR2 mediated-signaling existed in cultured MIO-M1 cells, we measured CCR3 and VEGFR2 protein and found both CCR3 and VEGFR2 were expressed. The expression of CCR3 and VEGFR2 was not affected by either or both CCL11 and VEGF (Fig 5A). In a parallel experiment, following incubation with CCL11 and VEGF, TUNEL+ cells were significantly increased in MIO-M1 cells pretreated with the CCR3i (100 nM) compared to control (DMSO). However, CCR3i did not increase TUNEL+ cells in response to CCL11 alone and decreased TUNEL+ cells when cells were incubated with VEGF alone (Fig 5B and 5C). In the same cell lysates, there was also no effect on cleaved caspase-3 by CCR3i-treatment compared to control (Fig 5D). However, in cells treated with CCL11 and VEGF or VEGF alone, CCR3i significantly decreased anti-apoptotic, BCL2 (Fig 5E and 5F), and increased AIF (Fig 5E and 5G) compared to control, whereas MIO-M1 cells incubated with either CCL11 or VEGF alone had decreased AIF protein compared to control (Fig 5E and 5G). Müller cells are known to express neurotrophic factors that are important for retinal neural and glial survival [23]. We determined if the induced apoptosis of MIO-M1 cells was associated with changes in the expression of the important Müller cell neurotrophic factor, brain-derived neurotrophic factor (BDNF). BDNF was measured in MIO-M1 lysates from all conditions. BDNF was significantly reduced following CCR3i pretreatment and exposure to CCL11 and VEGF, but not to CCL11 or VEGF treatment alone (Fig 5H and 5I). These findings present a scenario in which CCR3i reduced BDNF expression in Müller cells only following exposure to both CCL11 and VEGF and suggest that an interaction between the factors, perhaps through an adaptor protein, increased Müller cell susceptibility to apoptosis when exposed to CCR3 inhibition. VEGF is produced by Müller cells [19,24]; therefore, we measured VEGF expression in the same cell lysates. VEGF protein was not affected by treatment with CCR3i compared to control (S2A Fig), and this provided evidence that VEGF was not involved in CCR3i-induced MIO-M1 cell death, in agreement to our findings *in vivo* (Fig 4). Since inflammatary signaling contributes to laser-induced CNV, we determined if CCR3i-induced MIO-M1 cell death occurred through pyrotosis. We measured caspase 1 and cleaved caspase 1 (p20), which regulates pyrotosis, and did not detect p20 in cell lysates from any treatment (S2B Fig). Altogether, these data suggest that in the presence of CCL11 and VEGF, two factors that are increased in neovascular AMD, CCR3 inhibition induces Műller cell apoptosis and reduces the expression of the neurotrophic factor, BDNF.

### CCR3 Inhibition in RPE/choroid and cultured human CECs reduces VEGF signaling without inducing cell death

**Fig 6:**
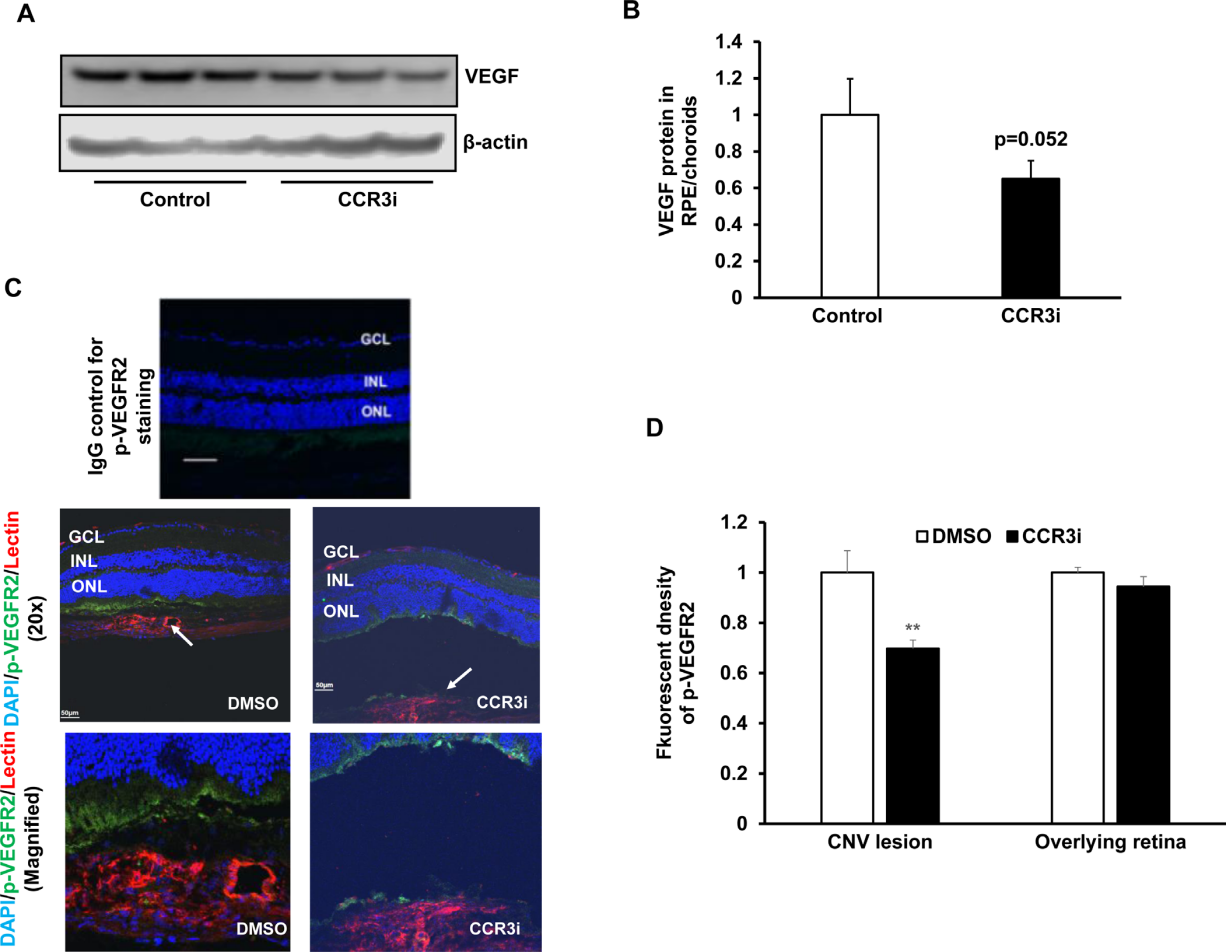
Inhibition of CCR3 inhibits VEGF signaling in RPE/choroids. (A) Western blots and (B) quantification of VEGF in RPE/choroids from 7 days after laser in control DMSO or CCR3i treated eyes (p = 0.052 *vs*. Control). (C) Immunostaining of phosphorylated VEGFR2 (p-VEGFR2) and (D) integrated density of p-VEGFR2 staining on retinal/RPE/choroidal sections from 7 days after laser in DMSO or CCR3i treated eyes (*p<0.05 *vs*. DMSO).

**Fig 7:**
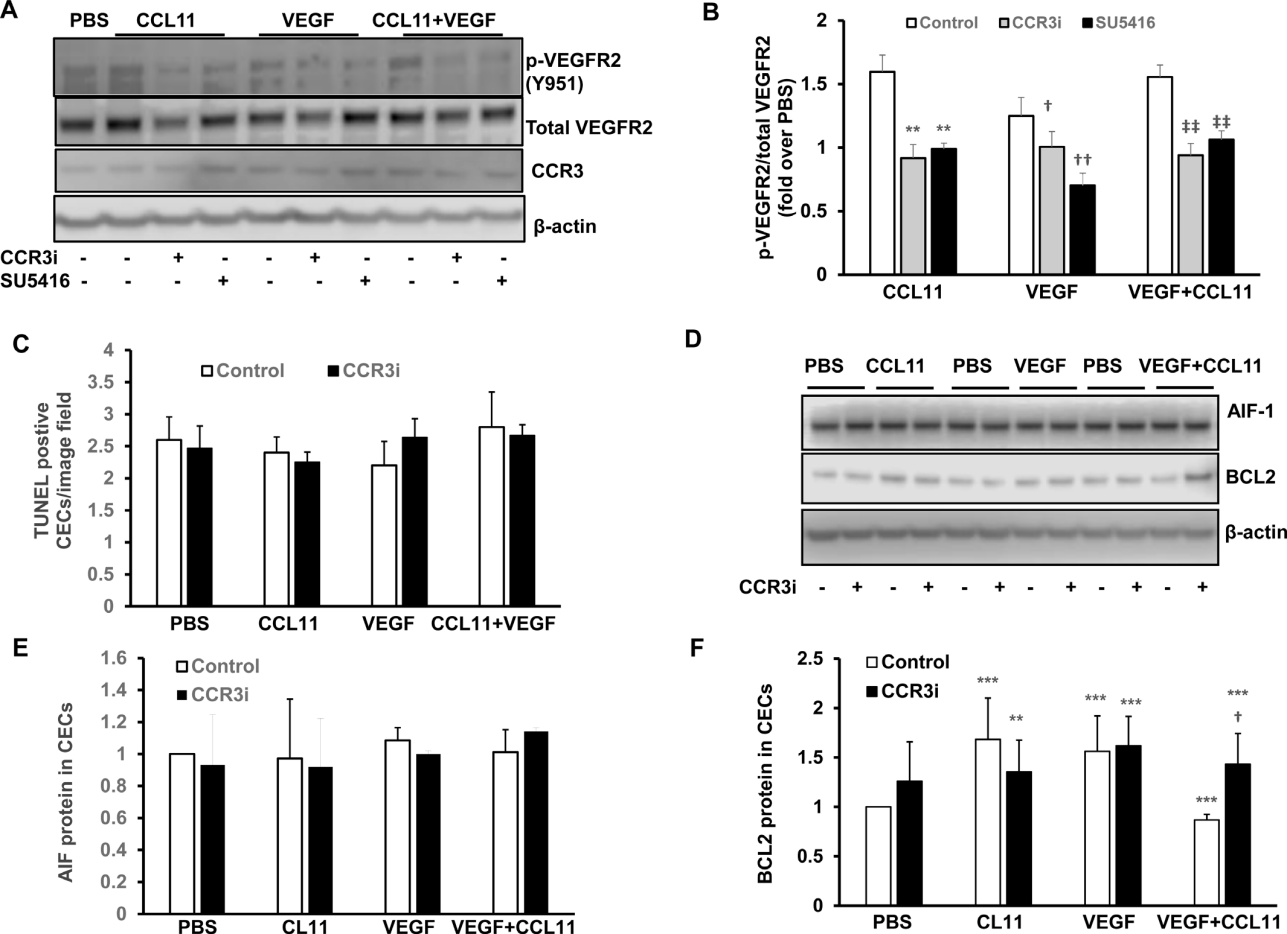
Inhibition of CCR3 in cultured human CECS reduces VEGF signaling without inducing cell death. In cultured human CECs treated with PBS or CCL11, VEGF or CCL11 and VEGF with or without pretreatment with CCR3i or control, (A) western blots of phosphorylated VEGFR2 (p-VEGFR2) and CCR3 and (B) quanfication of p-VEGFR2 (pretreatment with SU5416 was used as a control for inhibition of VEGFR2 activation) (**p<0.01, ***p<0.001 *vs*. Control of CCL11; ^†^p<0.05, ^††^p<0.01 *vs*. Control of VEGF; ^‡‡^p<0.01 *vs*. Control of CCL11 and VEGF). (C) Quantification of TUNEL+ cells. (D) Western blots and quantification of densitometry of (F) AIF and (F) BCL2 (**p<0.01, ***p<0.001 *vs*. Control of PBS; ^†^p<0.05 *vs*. Control of CCL11 and VEGF).

We previously reported that CCR3 interacted with VEGFR2 to increase CEC migration. CCR3 is a G-protein coupled receptor (GPCR), and GPCRs can transactivate receptor protein kinases. To obtain evidence as to whether CCR3 transactivated VEGFR2 signaling in vivo, we measured VEGF protein by western blot and immunolabeled retinal/RPE/choroidal cryosections for phosphorylated VEGFR2 (p-VEGFR2) 7 days following laser to induce CNV. As shown in Fig 6, compared to control, CCR3i treatment caused an insignificant reduction in VEGF protein in RPE/choroidal lysates (Fig 6A and 6B) but significantly reduced fluorescent density of p-VEGFR2 staining of lectin stained CNV lesions (Fig 6C and 6D). This evidence supported the thinking that CCR3 transactivation of VEGFR2 had been inhibited by CCR3i. There was no effect from the CCR3i on retinal p-VEGFR2 overlying CNV lesions (Fig 6D). We then measured p-VEGFR2 in cultured human CECs pretreated with CCR3i and exposed to CCL11 or/and VEGF. The VEGFR2 inhibitor, SU5416, was used to inhibit VEGFR2 phosphorylation in a comparison group. As shown in Fig 7A and 7B, p-VEGFR2 was increased by either CCL11 or VEGF treatment alone or combined treatment with CCL11 and VEGF, and this activation was inhibited by SU5416 or by CCR3i pretreatment, without affecting CCR3 expression. These data support the hypothesis that CCR3 transactivates VEGFR2 signaling.

We then determined the mechanism of angiogenic inhibition by CCR3i. Compared to DMSO control, CCR3i did not increase TUNEL+ cells (Fig 7C) or AIF protein in CEC lysates from cells treated with VEGF and CCL11 (Fig 7D and 7E), but increased the anti-apoptotic protein, BCL2, in the same CEC lysates (Fig 7D and 7F). These data do not support direct cell death as a result of CCR3i, but instead suggest that inhibition of CCR3 by CCR3i in CECs effectively reduced VEGF signaling without inhibiting cell survival. Increased TUNEL+ cells colabeled with lectin at CNV lesions (S1D Fig) may be leukocytes since isolectin stains both endothelial cells and leukocytes.

## Discussion

There is evidence that inhibition of the G-protein coupled receptor, CCR3, inhibits CNV, such as what occurs in neovascular AMD [6]. In this study, we sought to further understand the relationships between the CCR3 and VEGFR2 signaling pathways and to test the hypothesis that CCR3 inhibition caused cell death to CECs, activated to migrate and become CNV. We found that CCR3 inhibition alone did not induce CEC death, but in the setting of laser-induced CNV, CCR3 inhibition increased TUNEL+ cells within CNV lesions as well as in the retina overlying CNV lesions through mechanisms that appeared caspase 3 independent. It is possible that induced apoptosis was missed in lasered lesions when the entire retina was analyzed by western blot, but at two time points, we were still unable to find evidence of cleaved caspase-3 expression. We studied the effects of CCR3 inhibition on cultured CECs exposed to two factors that are implicated in the pathophysiology of neovascular AMD [6,7,25] and in laser-induced CNV, namely, CCL11 and VEGF. Compared to control, exposure to CCL11 and VEGF at doses previously shown to activate CECs failed to induce signs of cell death, apoptosis or pyroptosis measured by TUNEL labeling or western blots of cleaved caspase-3, cleaved caspase-1, BCL2 or AIF. When CECs were pretreated with the CCR3i and exposed to CCL11 and VEGF, there was an increase in BCL2 expression compared to CECs pretreated with vehicle control. Therefore, these data did not support the hypothesis that inhibition of CNV by CCR3i was due to direct CEC death or apoptosis. However, when CECs were pretreated with CCR3i or the VEGFR2 kinase inhibitor, SU5416, both inhibitors significantly reduced phosphorylated-VEGFR2 compared to control. The findings together with in vivo data support the thinking that CCR3 inhibition of CNV works by inhibiting VEGFR2 transactivation and not by directly causing CEC apoptosis or cell death.

Three days following laser, the CCR3i did not significantly affect CNV lesion width or height, but at day 7 the CCR3i reduced CNV volume and lesion width, but not height. VEGF was previously shown to be increased in the retina 1 day after laser-induced injury and to peak at day 3 [17]. Also, CCR3 did not significantly affect VEGF expression in the area of CNV [6], but affected VEGFR2 signaling in endothelial cells within CNV lesions. These findings support the thinking that in the first 3 days following laser injury, VEGF is a dominant angiogenic signaling mechanism, and that later CCR3 activation sustains neovascularization via transactivation of VEGFR2, as supported by data that CCR3i reduced CNV width and p-VEGFR2 seven days after laser.

Retinal thickness measured by OCT and on histologic sections was consistent throughout the retina and in the inner and outer nuclear layers and was no different between eyes treated with the intravitreal CCR3i or control supporting the thinking that CCR3 inhibition did not have detrimental effects on retinal morphology at the time points tested (data not shown). Therefore, in the short-term, inhibition of CCR3 did not show obvious detrimental effects on retinal structure. This evidence supports other studies in the literature that evaluated electroretinography (ERG) of eyes treated with CCR3i and found no safety issues following laser in the CNV model [6]. However these studies do not address long-term safety or potential harmful effects on certain cell types.

CNV is known to include a mixture of cell types, such as glia, RPE cells, endothelial cells, microglia and macrophages. We were unable to identify the TUNEL+ cells overlying CNV lesions by colabeling for several possible cell markers. Dying cells lose characteristic cell markers [26], and we recognize that the TUNEL+ cells may represent a mixture of cell types. Based on the literature, Műller cells have been shown to migrate in retinal degenerations, including in neovascular AMD [27,28]. Also, co-immunolabeling of CCR3 and Műller cell marker, glutamine synthetase, was demonstrated in retinal sections from non-lasered eyes. Therefore, we were interested in knowing if Műller cells were affected by CCR3i. Using cultured human MIO-M1 cells exposed to VEGF and CCL11 and treated with the CCR3i, we found a significant reduction in BCL2 and an increase in AIF compared to control, providing evidence that Műller cells may be susceptible to apoptosis by CCR3 inhibition when in the presence of CCL11 and VEGF. As an additional study to assess the effect of the CCR3i on Műller cells, MIO-M1 cells treated with CCR3i were found to have reduced BDNF. AIF-induced apoptosis was not noted in only CCL11 or only VEGF treated cells suggesting a need for the interaction between the signaling pathways. Both CCL11 and VEGF have increased expression in AMD. We postulate that VEGFR2 is transactivated by CCR3 in AMD and that inhibition of CCR3 also affects Műller cell survival. Although our studies were performed on cultured human Műller cells, the findings at least support further study and suggest caution and careful long-term studies if CCR3 inhibitors are proposed in human AMD. One option may be CEC targeted treatment to reduce potential adverse events on Műller cells, which are critical for retinal neural survival.

## Supporting Information

S1 Fig (A) TUNEL staining and (B) quantification of TUNEL+ cells in retinal cryosections from Non-lasered eyes 7 days after intravitreal injections of DMSO control or CCR3i. (C) TUNEL staining in isolectin stained retinal cryosections 7 days after laser. (D) Quantification of TUNEL+ cells in retina overlying CNV lesions from mouse eyes injected 7 days earlier with either IgG control or CCR3Ab following laser (1 μg in 1 μL; ***p<0.001 *vs*. IgG).
(TIF)

S2 Fig (A) Western blots and quantification of VEGF and (B) western blots of caspase-1 in MIO-M1 cells pretreated with CCR3i (100nM) or DMSO control and exposed to PBS, CCL11, VEGF, or CCL11+VEGF.
(TIF)
