# Supplementary figures and images for: Retinal Inhibition of CCR3 Induces Retinal Cell Death in a Murine Model of Choroidal Neovascularization
Source: PLoS One. 2016 Jun 16;11(6):e0157748. doi: 10.1371/journal.pone.0157748 (PMC4911089; doi:10.1371/journal.pone.0157748)

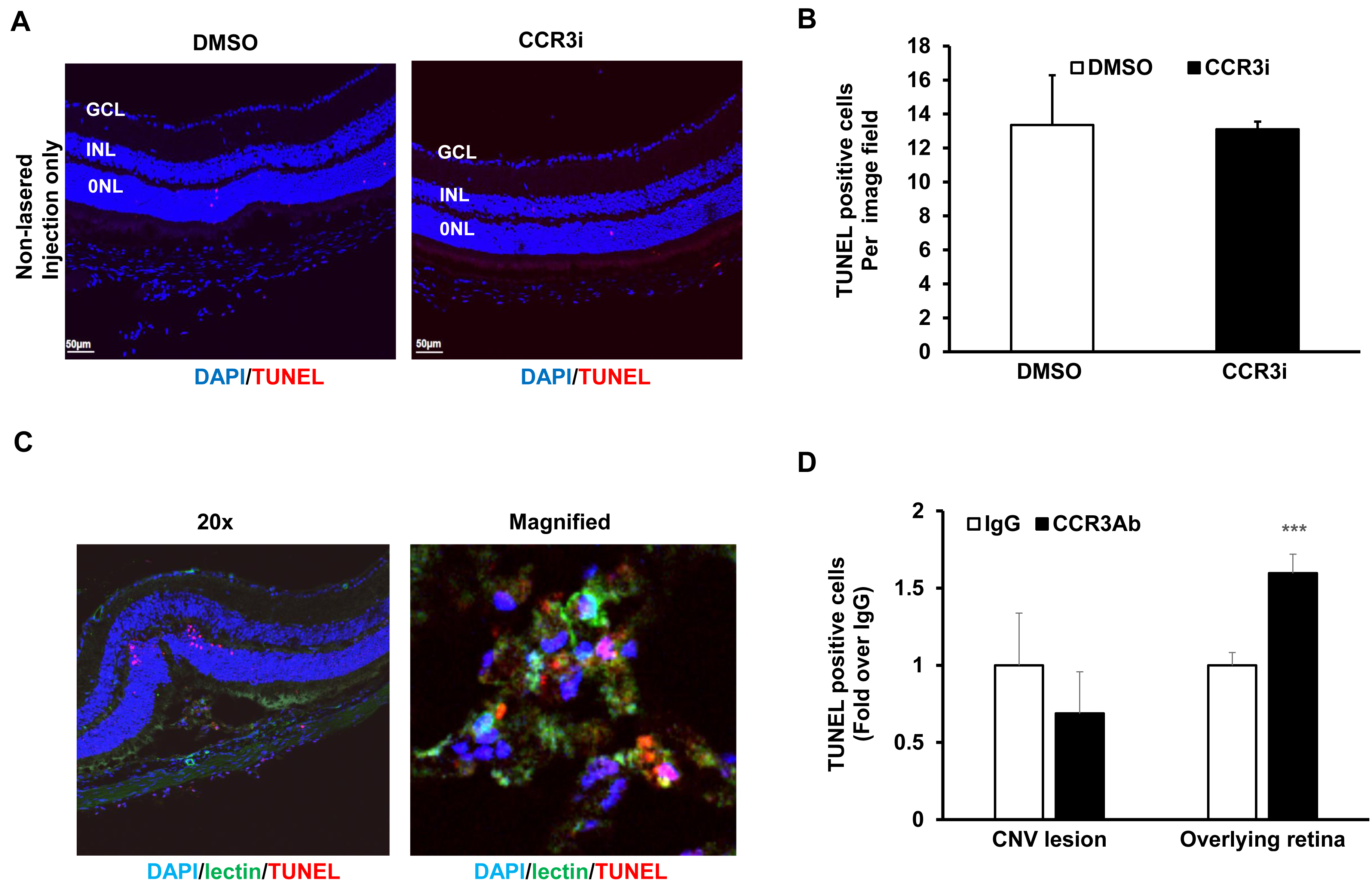

Supplement: S1 Fig — (A) TUNEL staining and (B) quantification of TUNEL+ cells in retinal cryosections from Non-lasered eyes 7 days after intravitreal injections of DMSO control or CCR3i. (C) TUNEL staining in isolectin stained retinal cryosections 7 days after laser. (D) Quantification of TUNEL+ cells in retina overlying CNV lesions from mouse eyes injected 7 days earlier with either IgG control or CCR3Ab following laser (1 μg in 1 μL; ***p<0.001 vs. IgG). (TIF) [file pone.0157748.s001.tif]

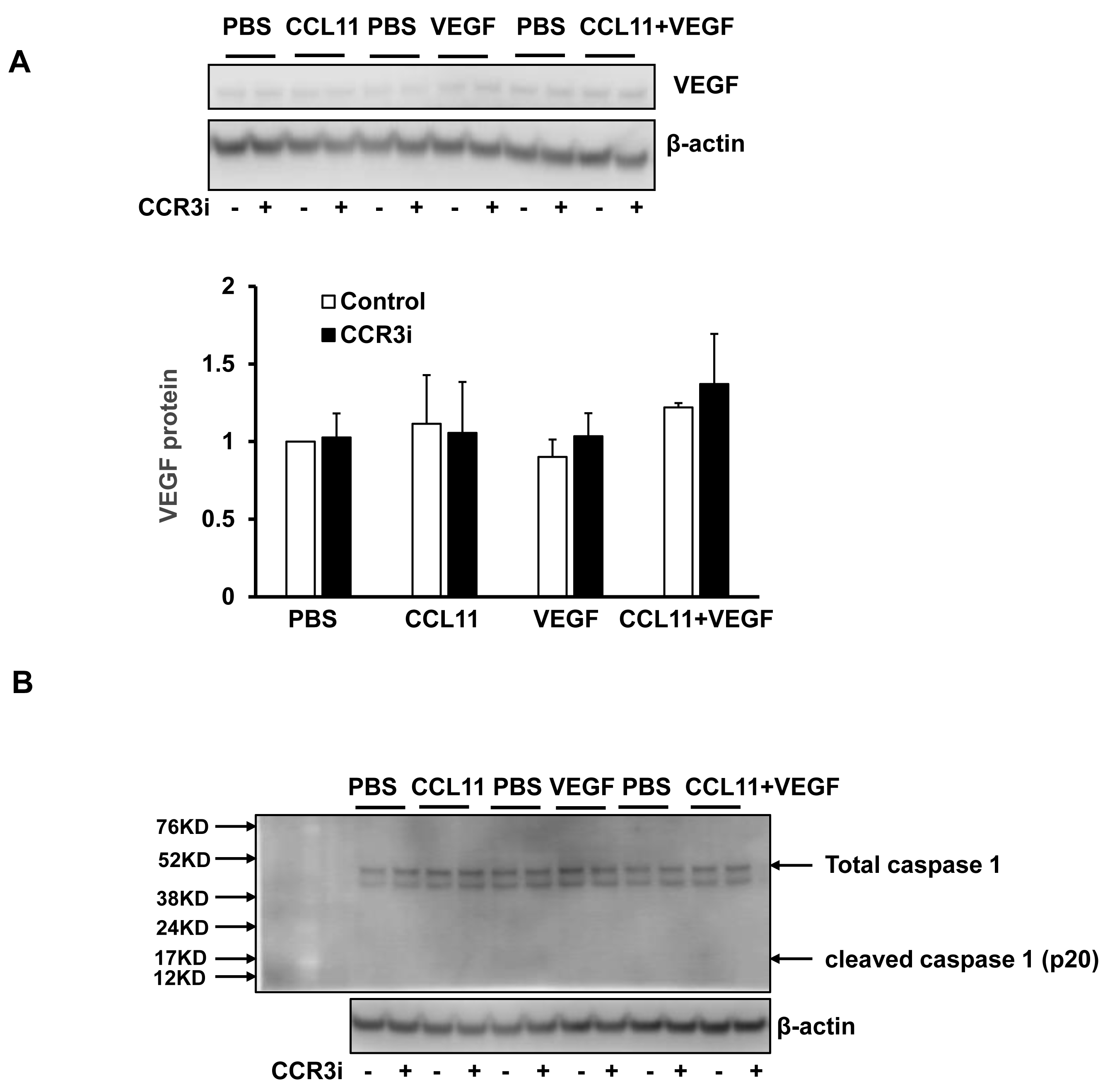

Supplement: S2 Fig — (A) Western blots and quantification of VEGF and (B) western blots of caspase-1 in MIO-M1 cells pretreated with CCR3i (100nM) or DMSO control and exposed to PBS, CCL11, VEGF, or CCL11+VEGF. (TIF) [file pone.0157748.s002.tif]
